# Commentary: CO2 pneumoperitoneum clinical study pitfalls

**DOI:** 10.3389/fped.2025.1587078

**Published:** 2025-04-17

**Authors:** Natalya L. Davydenko, Kaldybay S. Idrissov, Lyazzat E. Alikanova, Victor V. Kazenashev, Ospan A. Mynbaev

**Affiliations:** ^1^ Gynecology Department, Central Clinical Hospital «RZD-Medicine», Moscow, Russia; ^2^ General Practitioner Department, South Kazakhstan Medical Academy, Shymkent, Kazakhstan; ^3^ General Practitioner Department N1, Shymkent Campus, Khoja Akhmet Yassawi International Kazakh-Turkish University, Turkistan, Kazakhstan; ^4^ Department of Obstetrics, Gynecology and Reproductive Medicine, Faculty of General Medicine, Russian University of Medicine, Moscow, Russia; ^5^ The New European Surgical Academy, Berlin, Germany

**Keywords:** CO2 pneumoperitoneum, intraperitoneal pressure, neonatal laparoscopic surgery, blood gas acid-base changes, carboxemia, metabolic acidemia, oxidative stress, acute inflammatory cytokines

A Commentary on Application of different CO2 pneumoperitoneum pressure in laparoscopic pyeloplasty for infants with ureteropelvic junction obstruction By Peng Y, Zhu M, Chen C (2024). Front. Pediatr. 12:1380985. doi: 10.3389/fped.2024.1380985

We read with great interest the paper by Peng et al. (1) that studied different CO2 pneumoperitoneum pressures during laparoscopic pyeloplasty in infants. It is a critical study with essential findings for clinicians, especially pediatric surgeons, due to increased surgical activities in neonatology using laparoscopic and robot-assisted surgery when higher CO2 pneumoperitoneum pressure is applied.

In this study (1), the authors demonstrated significant changes in blood gas acid-base parameters, including pH, arterial partial pressure of carbon dioxide (PaCO2), actual base excess (ABE), and standard base excess (SBE) at the point of 30 min after insufflation in comparison with 5 min before insufflation in both groups of patients with CO2 pneumoperitoneum pressures at five and eight mmHg in the abdominal cavity. These changes were accompanied by increased parameters of various stress indexes, inflammatory cytokines, and oxidative stress markers, including cortisol, epinephrine, interleukin (IL)-6, tumor necrosis factor-alpha (TNF-α), serum malondialdehyde (MDA) and reduction of superoxide dismutase (SOD) at this sampling point (30 min after insufflation). All these changes were more pronounced in patients with the CO2 pneumoperitoneum pressure at eight mmHg in the abdominal cavity than at five mmHg, emphasizing extreme caution in applying higher CO2 pneumoperitoneum pressures for infants.

**Figure 1:**
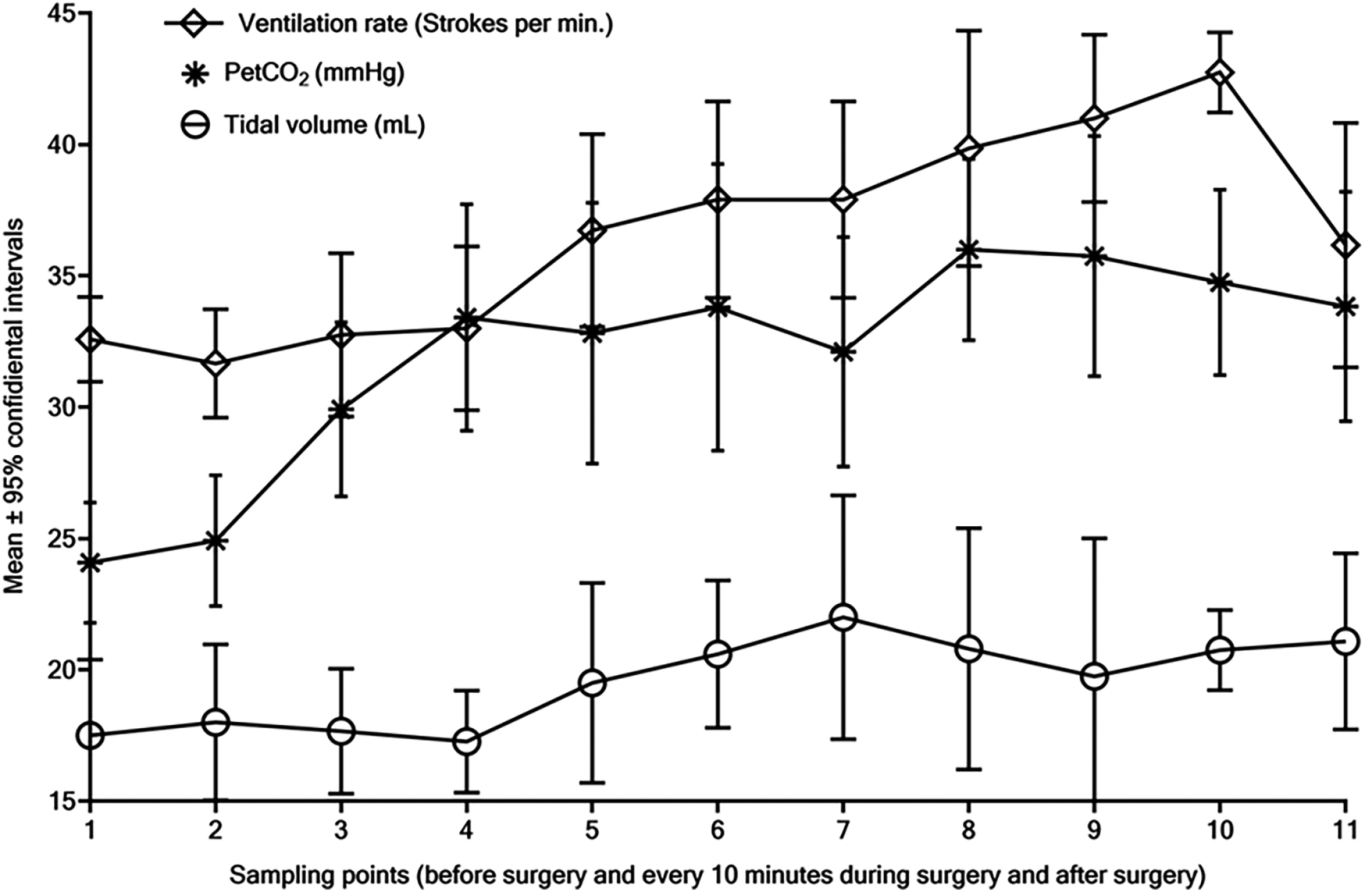
Dynamic changes of the end-tidal CO2 (PetCO2, mmHg), ventilation rate (strokes per minute), and tidal volume (ml) during laparoscopic surgical procedures with CO2 pneumoperitoneum at 7–8 mmHg due to ovarian tumors in 12 newborns. All samples were collected at the time of induction and incision, then every 10 min during surgery, after surgery for one and a half hours, subsequently at the eleven-time points (1–11), and all findings were presented as a mean with 95% confidential intervals.

These changes in blood gas acid-base parameters (1) correspond with our results from experimental (2) and clinical (3) studies. We observed increased PaCO2 and decreased pH in the arterial blood of rabbits depending on CO2 pneumoperitoneum pressure and duration of the carbon dioxide insufflation (2). Analogously, the end-tidal CO2 (PetCO2) concentration was substantially increased during laparoscopic surgery with CO2-pneumoperitoneum at 7–9 mmHg in 12 newborns due to ovarian tumors (3). Further elevation of the PetCO2 was controlled by mild hyperventilation with increased ventilation rate without changes of tidal volume in these newborns (Figure 1). In addition; we observed increased systolic/diastolic arterial blood pressures and the peak of respiratory pressure in these newborns (3). Meanwhile, cardiac output decreased, and heart rate, urine output, and skin temperature remained stable (3). Analogously, in our experimental studies in rabbit models, mechanical lung ventilation was used with increased tidal volume to prevent hypercarboxemia and acidosis compared with severe changes of acid-base equilibrium parameters in animals with lower tidal volume ventilation mode (2).

Unfortunately, the authors (1) did not include capnography findings, such as PetCO2 and other lung function parameters during laparoscopic surgery. They also did not describe a mechanical lung ventilation mode to prevent expected CO2 pneumoperitoneum adverse effects and complications in their vulnerable patients (1).

It is well known that anesthesiological routine procedures during laparoscopic surgery with CO2 pneumoperitoneum included monitoring of PetCO2 with correction of minute ventilation volume by increased ventilation rates or tidal volume to prevent hypercarboxemia, acidosis, and other intraoperative CO2 accumulation side effects and post-surgical complications. Subsequently, in this study (1), controlled under PetCO2, different lung ventilation modes should be applied depending on CO2 pneumoperitoneum pressure levels to reduce the side effects of CO2 accumulation in their patients.

Generally, such clinical studies should consider all surgical and anesthesiological factors associated with the surgical approach, i.e., laparoscopy and robot-assisted surgery. The study (1) aimed to determine the impact of different CO2 pneumoperitoneum pressures on the physiological function of infants. Therefore, the absence of information concerning anesthesiology management with PetCO2 monitoring and lung ventilation modes is a significant oversight in this study (1), underscoring the importance of comprehensive studies to ensure the safety and well-being of vulnerable pediatric patients.
